# An Experimental and Critical Inquiry into the Nature and Treatment of Wounds of the Intestines

**Published:** 1843-12-09

**Authors:** 


*An Experimental and Critical Inquiry into the Nature and Treatment of Wounds of the Intestines. Illustrated by Engravings.* By Samuel D. Gross, M. D., Professor of Surgery in the Louisville Medical Institute, etc. etc. etc. Louisville: Prentice & Weissinger. 1843. 8vo. pp. 219.

"A monograph on wounds of the intestines has long been an acknowledged desideratum in our surgical literature. The work of Mr. Travers, the only work of the kind in the English language, has been out of print for nearly a quarter of a century, and hence the only information accessible to practitioners, especially to those of the United States, is such as is to be found in the various periodicals of the day, in the transactions of societies, or in our systematic treatises on surgery. The latter, unfortunately, contain little, if any thing, that is worthy of reliance; they enter into no details, and some of them do not even allude to the subject; a circumstance so much the more surprising when we reflect upon the importance of these injuries, and the attention which has been bestowed upon them by some of the most respectable members of the profession. In the following pages an attempt has been made to supply this deficiency, by exhibiting a connected view of the subject, embracing an account of the results of my own researches, and of those who have preceded me in the same field of inquiry.

The experiments which form the basis of the present treatise, were commenced in the spring of 1841, and continued, with various intermissions, until a few months ago. The object was, in the first place, to inquire into the process employed by nature in repairing wounds of the intestinal tube; and secondly, and more particularly, to determine if possible, the value of the more important methods of treatment recommended by surgeons from the time of Ramdohr down to our own. The experiments, amounting altogether to upwards of seventy in number, were performed exclusively upon the dog, as the most eligible animal that could be procured for the purpose. The wound was generally made in the small bowel, not only because it is the more accessible portion of the alimentary tube, but because it is more liable, when thus injured, to become the seat of fæcal effusion, and also, perhaps, of high inflammation. The results are stated, in every instance, with as much brevity as is consistent with a work of this description."—(*Preface*, pp. v.—vi.)

The first chapter treats of the Nature of Wounds of the Intestines, including, besides, the anatomical structure of the contents of the abdominal cavity, the symptoms, diagnosis, prognosis, and mode of reparation. The second chapter is an extended one, and the Treatment of Wounds of the intestines is discussed in all its bearings. Professor Gross thinks that the fatality of penetrating wounds of the abdomen has been much exaggerated. Wounds of the peritoneum have been a sort of bugbear with surgeons, not so much from what they have seen, but from what they have heard. Our author thinks that where the wound of the intestine is of any size, the external wound should be dilated, if necessary, and the intestinal lesion secured by means of sutures, in order that subsequent effusion may be prevented. When the wound in the bowel is not more than the third or fourth of an inch, such, of course, would be unjustifiable.

The space occupied by the Clinical Lectures in our present number does not enable us to do justice to Dr. Gross' work, and we have already postponed our notice of it too long to admit of further delay. It displays much labour and research. The author has sustained his views and those of others by a direct appeal to experiment. We consider it a valuable contribution to our literature, and as worthy of the high reputation of the author. We intend, at more leisure, to recur to it, and until then, recommend it to our readers; for similar exertions on behalf of the profession, should be met with a corresponding spirit.

